# Clinical interpretation of cell-based non-invasive prenatal testing for monogenic disorders including repeat expansion disorders: potentials and pitfalls

**DOI:** 10.3389/fgene.2023.1188472

**Published:** 2023-09-27

**Authors:** Line Dahl Jeppesen, Lotte Hatt, Ripudaman Singh, Palle Schelde, Katarina Ravn, Christian Liebst Toft, Maria Bach Laursen, Jakob Hedegaard, Inga Baasch Christensen, Bolette Hestbek Nicolaisen, Lotte Andreasen, Lars Henning Pedersen, Ida Vogel, Dorte Launholt Lildballe

**Affiliations:** ^1^ ARCEDI Biotech, Vejle, Denmark; ^2^ Center for Fetal Diagnostics, Aarhus University, Aarhus, Denmark; ^3^ Department of Molecular Diagnostics, Aalborg University Hospital, Aalborg, Denmark; ^4^ Center for Preimplantation Genetic Testing, Aalborg University Hospital, Aalborg, Denmark; ^5^ Department of Clinical Genetics, Aarhus University Hospital, Aarhus, Denmark; ^6^ Department of Gynecology and Obstetrics, Aarhus University Hospital, Aarhus, Denmark; ^7^ Department of Clinical Medicine, Aarhus University, Aarhus, Denmark; ^8^ Department of Biomedicine, Aarhus University, Aarhus, Denmark; ^9^ Department of Molecular Medicine, Aarhus University Hospital, Aarhus, Denmark

**Keywords:** noninvasive prenatal testing, fetal cells, extravillous trophoblasts, monogenic disorders, repeat expansion disorders, clinical interpretation

## Abstract

**Introduction:** Circulating fetal cells isolated from maternal blood can be used for prenatal testing, representing a safe alternative to invasive testing. The present study investigated the potential of cell-based noninvasive prenatal testing (NIPT) for diagnosing monogenic disorders dependent on the mode of inheritance.

**Methods:** Maternal blood samples were collected from women opting for prenatal diagnostics for specific monogenic disorders (*N* = 7). Fetal trophoblasts were enriched and stained using magnetic activated cell sorting and isolated by fluorescens activated single-cell sorting. Individual cells were subject to whole genome amplification, and cells of fetal origin were identified by DNA-profiling using short tandem repeat markers. The amplified fetal DNA was input for genetic testing for autosomal dominant-, autosomal recessive-, X-linked and repeat expansion disorders by direct variant analysis and haplotyping. The cell-based NIPT results were compared with those of invasive testing.

**Results:** In two cases at risk of skeletal dysplasia, caused by variants in the *FGFR3* gene (autosomal dominant disorders), cell-based NIPT correctly stated an affected fetus, but allelic dropout of the normal alleles were observed in both cases. Cell-based NIPT gave an accurate result in two cases at risk of autosomal recessive disorders, where the parents carried either different diastrophic dysplasia causing variants in the *SLC26A2* gene or the same cystic fibrosis disease-causing variant in the *CFTR* gene. Cell-based NIPT accurately identified an affected male fetus in a pregnancy at risk of Duchenne muscular dystrophy (*DMD* gene, X-linked recessive disorders). In two cases at risk of the myotonic dystrophy type 1 (*DMPK* gene, repeat expansion disorder), cell-based NIPT correctly detected an affected and an unaffected fetus, respectively.

**Discussion:** Circulating fetal cells can be used to detect both maternally- and paternally inherited monogenic disorders irrespective of the type of variant, however, the risk of allelic dropout must be considered. We conclude that the clinical interpretation of the cell-based NIPT result thus varies depending on the disorders’ mode of inheritance.

## 1 Introduction

Prenatal diagnosis of monogenic disorders requires invasive tissue sampling of chorionic villi (CVS) or amniotic fluid (AC). Invasive tissue sampling, however, causes discomfort for the pregnant woman and possesses a small risk of induced abortion (Wulff et al., 2016; Salomon et al., 2019). Noninvasive prenatal tests (NIPT) are therefore often preferred among pregnant women as a risk-free alternative (Hill et al., 2014; Hill et al., 2016; Lund et al., 2018; Mohan et al., 2022; Toft et al., 2022). Fetal cell-free DNA in maternal plasma has proven to be a useful source of fetal DNA for the diagnosis of autosomal dominant (AD) disorders caused by *de novo* or paternally-inherited variants (Chitty et al., 2011; Scotchman et al., 2020b), but has not yet been widely implemented. The diagnosis of maternally inherited AD disorders, autosomal recessive (AR) and X-linked (XL) disorders using cell-free DNA are further challenged by two matters: The high amount of contaminating maternal cell-free DNA compared to fetal cell-free DNA and the method’s dependency on paternal samples for dosage-based techniques and haplotyping (Hanson et al., 2022). Therefore, prenatal testing of monogenic disorders using cell-free DNA can only be provided on a patient- and disease-specific basis (Scotchman et al., 2020a).

Cell-based NIPT is based on the isolation and genetic analysis of fetal or placental cells harvested from the maternal circulation (Hatt et al., 2014; Singh et al., 2017; Ravn et al., 2020). In this study, circulating extravillous trophoblasts (EVTs) derived from the tip of the anchoring chorionic villous were enriched using antibodies targeting a combination of mesenchymal and epithelial markers. These cells hold an uncontaminated intact fetal genome, which allows for direct variant detection. Thus, in addition to NIPT for paternal and *de novo* variants, the cell-based approach holds the potential to provide noninvasive prenatal diagnosis of maternally inherited AD disorders, AR and XL disorders. Furthermore, the intact fetal genome may allow for direct variant analysis of trinucleotide repeat expansions, in which anticipation is a phenomenon whereby the symptoms of a repeat expansion disorders have earlier onset and become more severe when passed on to the next-generation (Mirkin, 2007).

This study contributes to the growing area of research exploring cell-based NIPT for monogenic disorders (Saker et al., 2006; Mouawia et al., 2012; Chang et al., 2021; Jeppesen et al., 2021; Zhuo et al., 2021). Recently, we showed how circulating EVTs could offer prenatal screening for cystic fibrosis early in pregnancy without need for partner- or proband samples (Jeppesen et al., 2023), and be used to verify the fetal status following preimplantation genetic testing for monogenic disorders (Toft et al., 2021). Here, we present a series of seven pregnancies at risk of monogenic AD, AR, XL, or repeat expansion disorders to highlight the feasibility of cell-based NIPT for detection of different types of both paternally- and maternally inherited variants.

## 2 Materials and methods

### 2.1 Patient recruitment

Pregnant women were recruited when opting for prenatal diagnosis for a monogenic disorder following genetic counseling at the Department of Clinical Genetics, Aarhus University Hospital. They received oral and written information about the study and subsequently gave written informed consent to participate. The study was approved by the Central Denmark Region Committee on Health Research Ethics (1-10-72-225-19). The cell-based NIPT results were compared with those of prenatal diagnostics performed on invasive tissue samples at the Department of Clinical Genetics at Aarhus University Hospital, Denmark.

### 2.2 Blood sampling and preparation

30–60 mL of peripheral blood was collected in Cell-Free DNA BCT tubes (Streck laboratories, US) before CVS. Blood was processed according to the procedure for EVT enrichment previously described by (Hatt et al., 2014). In brief, the nucleated cells were fixed and permeabilized, red blood cells were lysed, and the EVTs were enriched by magnetic activated cell sorting (Miltenyi Biotec, Germany) using microbead conjugated monoclonal mouse anti-human CD105 and CD141 antibodies followed by immunostaining using a cocktail of fluorophore conjugated monoclonal mouse antibodies targeting human cytokeratin expressed in EVTs, as well as CD14 and CD45.

### 2.3 Extravillous trophoblast isolation

Fluorescence activated cell sorting (FACS) was used for single cell isolation using a FACS Melody (BD Biosciences, US). Candidate cells were isolated from a CK^+^/CD14^-^/CD45^-^ sorting gate and the genomes were amplified using PicoPLEX Single Cell WGA Kit v3 (Takara Bio, US). The origin of the isolated cells was determined by short-tandem repeat (STR) marker analysis using GlobalFiler™ PCR Amplification Kit (Thermo Fisher Scientific, US), as previously described (Hatt et al., 2020). Cells were classified as fetal if non-maternal alleles were observed.

### 2.4 FGFR3-related disorders (AD skeletal dysplasias, common variants)

For the detection of *FGFR3*-related disorders, amplicons were designed to cover the *FGFR3* variant of case 1a (NM_000142.4(*FGFR3*):c.1620C>G and case 1b (NM_000142.4(*FGFR3*):c.1138G>A). One µL single-cell WGA-DNA product from cells of fetal origin was used as input for PCR amplification using 5 U AmpliTaq Gold™ 360 DNA Polymerase with 10X AmpliTaq Gold^®^ 360 Buffer, 4 μL GC Enhancer (Thermo Fisher Scientific, US), 1.5 mM MgCl_2_ (25 mM) and 2 mM dNTP (10 mM solution of dNTP containing 2.5 mM each of dATP, dCTP, dGTP and dTTP) in a final reaction volume of 25 µL. The forward- and reverse primers were added for a final concentration of 0.2–2 µM (Supplementary Table S1). Thermal cycling was performed with an initial denaturation at 95°C for 10 min followed by 33 cycles of 95°C for 30 s, primer specific annealing temperature for 30 s and 72°C for 45 s, and with a final extension at 72°C for 7 min before a hold at 4°C using a Veriti™ 96-Well Thermal Cycler (Thermo Fisher Scientific, US). The PCR products were analyzed by capillary electrophoresis using an ABI 3500 (Thermo Fisher Scientific, US) for size and purity. The reverse primers (2 pmol/μL) were used for Sanger Sequencing at the Department of Molecular Medicine (MOMA), Aarhus University Hospital, Denmark. Data analysis was performed using Sequencing Analysis Software 6 (Applied Biosystems, Thermo Fisher Scientific, US).

### 2.5 Diastrophic dysplasia (AR skeletal disorders, family-specific variants)

Primers were designed for two amplicons to cover the maternal (NM_000112.3(*SLC26A2*):c.-26 + 2T>C) and paternal (NM_000112.3(*SLC26A2*):c.1957T>A) variants (Supplementary Table S2). The PCR amplifications were run in a 25-µL reaction with the same PCR mix as for RP-PCR and with a final concentration of the forward and reverse primer of 1.0 µM of each. The template was 20 ng single-cell WGA-DNA, 20 ng WGA-DNA pool from all EVTs, or 0.5 ng gDNA. The PCR program was 10 min at 95°C, 33 cycles of 95°C for 30 s, 58°C (for *SLC26A2* c.-26 + 2T>C) or 59°C (for *SLC26A2* c.1957T>A) for 30 s and 72°C for 30 s, followed by 7 min final extension at 72°C and a hold at 4°C. Sanger sequencing was performed with *SLC26A2* c.-26 + 2T>C forward primer, and *SLC26A2* c.1957T>A reverse primer, at MOMA, Aarhus University Hospital, Denmark. Data analysis was done using Sequencing Analysis Software 6 (Applied Biosystems, Thermo Fisher Scientific, US).

### 2.6 Cystic fibrosis (AR multiorgan disorder, common variants)

Cystic fibrosis analysis was conducted as previously described using a pool of amplified DNA from fetus 1 and fetus 2, respectively (Jeppesen et al., 2023). In brief, ARMS-PCR was conducted using CFEU2v1 (Elucigene, UK) followed by fragment-length analysis using an Applied Biosystem 3,500 Genetic Analyzer (Thermo Fisher Scientific, US) using the manufacturers’ panel and bins.

### 2.7 Duchenne muscular dystrophy (XL muscular wasting disease, a family-specific variant)

A primer set was designed to cover the family specific variant NM_004006.2(*DMD*):c.4358_4359insAATA. The forward- and reverse primers were tagged with a universal M13-primers sequence for Sanger Sequencing in both directions (Supplementary Table S3). A FAM labeled forward primer was used for fragment length analysis using ABI 3500. The PCR was run in a 25-µL reaction volume with 1 X Q-solution, 1 X PCR buffer, 1 U of HotStarTaq DNA polymerase (Qiagen, DE), 0.2 mM dNTP and a final concentration of the forward and reverse primers of 0.5 uM each. The template was 1 µL of WGA-DNA from individual EVTs or a pool of WGA-DNA. Thermal cycling was performed with 15 min at 95°C followed by 35 cycles of 94°C for 1 min, 60°C for 30 s and 72°C for 1 min, and terminated by a final extension at 72°C for 10 min. Sanger Sequencing was conducted at the Department of Molecular Medicine, Aarhus University Hospital. Sanger sequencing data was accessed using Sequencing Analysis Software 6 (Applied Biosystems, Thermo Fisher Scientific, US).

### 2.8 Myotonic dystrophy type 1 (AD muscle wasting disorder caused by repeat expansion)

The *DMPK* repeat expansion analysis was conducted using repeat-primed PCR (RP-PCR) targeting the trinucleotide CTG-repeat expansion and a separate multiplex PCR targeting six short tandem repeats flanking the repeat region (Supplementary Table S4). The 3′ TP-PCR was conducted in a 25-µL reaction volume with 5 U AmpliTaq Gold™ 360 DNA Polymerase, 1x AmpliTaq Gold 360 PCR-buffer, 1.5 mM MgCl_2_, 1 × 360 GC Enhancer (Thermo Fisher Scientific, US) and a 10 mM solution of dNTP for a final concentration of 100 µM of each of dATP, dTTP, dCTP and dGTP. The primers were adopted from Lian et al., 2015; Lian et al., 2015) and the assay used 0.4 µM *DMPK* flanking (Fam-3′ R) and tail primer (3′ Tail) and 5-fold diluted triplet-primed primer (3′ TPF). The template was 1 µL of single-cell WGA-DNA or 1 ng maternal gDNA. Thermal cycling was performed using a Veriti™ 96-Well Thermal Cycler (Thermo Fisher Scientific, US) with the following program: Initial denaturation at 95°C for 10 min followed by 10 cycles of 94°C for 30 s, 60°C for 1 min, 72°C for 4 min followed by 25 cycles of 94°C for 30 s, 60°C for 1 min, 72°C for 1 min + 20 s/cycle, and a final extension for 10 min at 72°C before a final hold at 4°C. The PCR products were analyzed by capillary electrophoresis using an ABI 3500 (Thermo Fisher Scientific, US) by combining 3 µL of the PCR product with 9.6 µL Hi-Di™ Formamide (Thermo Fisher Scientific, US) and 0.4 µL GeneScan™ 600 LIZ™ Dye Size Standard (Thermo Fisher Scientific, US).

The primer positions for the six STR markers were adopted from Lian et al., 2017; Lian et al., 2017) and with a final concentration of 0.8–2.0 μmol/L for each primer. The PCR multiplex amplification was run in a 25-µL reaction volume using the same PCR mix as described for RP-PCR. The template for the STR analysis was a pool of WGA-DNA from EVTs or 1 ng maternal gDNA. The thermal cycling was performed with an initial denaturation for 10 min at 95°C followed by 26 cycles for WGA-DNA or 30 cycles for gDNA of 95°C for 30 s, 60°C for 1.5 min and 72°C for 1 min followed by final extension for 5 min at 72°C and 10 min at 60°C and hold at 4°C. Fragment analysis was conducted on an ABI 3500 (Thermo Fisher Scientific, US) using 3 µL PCR product, 9.6 µL Hi-Di™ Formamide (Thermo Fisher Scientific, US) and 0.4 µL GeneScan™ 600 LIZ™ Dye Size Standard (Thermo Fisher Scientific, US) and GeneMapper software (Thermo Fisher Scientific, US).

## 3 Results

Table 1 summaries the case stories and the results of cell-based NIPT compared to invasive testing.

**TABLE 1:** Case characteristics.

Case	Blood sampling GA (week+day)	Number of EVTs	Variant in affected parent(s)	Disease	Inheritance	Analysis method	Cell-based NIPT result	CVS results
1a	10 + 2	2	Paternal: NM_000142.5 (*FGFR3*):[c.1620C>G];[ = ]	Hypochondroplasia	AD	Sanger sequencing	Affected, ADO of normal allele	Affected
1b	16 + 5	1	Maternal: NM_000142.5(*FGFR3*):[c. 1138G>A];[ = ]	Achondroplasia	AD	Sanger sequencing	Affected, ADO of normal allele	Affected
2	12 + 3	9	Maternal: NM_000112.3(*SLC26A2*):[c.-26 + 2T>C];[ = ]	Diastrophic dysplasia	AR	Sanger sequencing	Affected (compound heterozygote)	Affected (compound heterozygote)
			Paternal: NM_000112.3 (*SLC26A2*):[c.1957T>A];[ = ]					
3	13 + 6	Dichorionic twins: fetus 1: 2, fetus 2: 3	Maternal and paternal: *CFTR*(NM_000492.3):[c.1521_1523del];[ = ]	Cystic fibrosis	AR	ARMS-PCR and fragment length analysis (CFEU2v1, Elucigene)	Fetus 1: unaffected carrier	Fetus 1: unaffected carrier
							Fetus 2: affected (homozygote for F508del)	Fetus 2: affected (homozygote for F508del)
4	13 + 2	2	Maternal: NM_004006.3(*DMD*): [c.4358_4359insAATA];[ = ]	Duchenne muscular dystrophy	XLR	Sanger sequencing	Male fetus, affected	Male fetus, affected
5a	12 + 6	8	Maternal: NM_004409.5(*DMPK*):c.*224CTG[normal allele];[>150]	Myotonic dystrophy type 1	Repeat expansion	Repeat-primed PCR and STR haplotyping	Affected, >150 CTG-repeats	Affected (5-CTG, >150-CTG)
5b	10 + 4	3	Maternal: NM_004409.5(*DMPK*):c.*224CTG[normal allele];[520]	Myotonic dystrophy type 1	Repeat expansion	Repeat-primed PCR and STR haplotyping	Unaffected, no repeat expansion, two normal alleles detected by one fully informative and one semi-informative STR marker	Unaffected (11-CTG, 12-CTG)

^‡^60 mL of blood, GA, gestational age; EVTs, extravillous trophoblasts; ADO, allelic dropout; AD, autosomal dominant; AR, autosomal recessive; XLR, X-linked recessive; STR, short tandem repeat.

### 3.1 *FGFR3*-related disorders (AD skeletal dysplasia, common variants)

**FIGURE 1:**
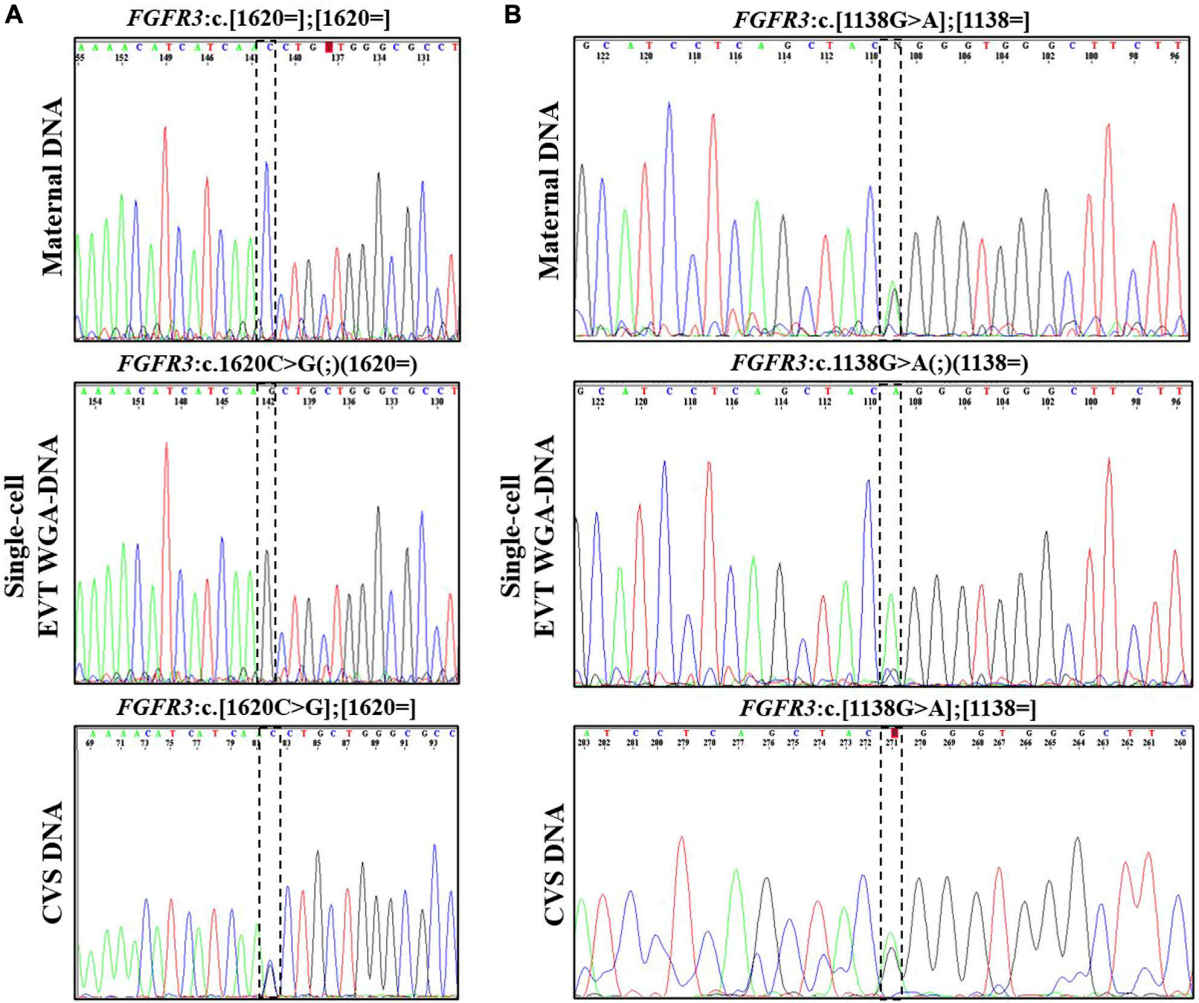
Cell-based NIPT for *FGFR3*-related disorder. **(A)** Case 1a Sanger sequencing result of maternal DNA and single-cell EVT WGA-for detection of the paternally inherited disease-causing *FGFR3* c.1620C>G variant. Allelic dropout was observed for the maternally inherited normal allele. The result suggest that the fetus is affected by autosomal dominant hypochondroplasia. **(B)** Case 1b Sanger sequencing result of maternal DNA, WGA-DNA of a single EVT and CVS for detection of the maternally inherited *FGFR3* c.1138G>A. Allelic dropout of the paternally inherited normal allele was observed. The result indicate that the fetus was affected by autosomal dominant achondroplasia.

In case 1a, the paternally inherited *FGFR3* c.1620C>G variant was detected by Sanger sequencing in two EVTs, indicating that the fetus was affected with hypochondroplasia. Allelic dropout (ADO), i.e., failure to detect one allele, was observed for the normal allele (ADO: 50%). The Sanger sequencing results are shown in Figure 1A.

In case 1b, the mother was affected by achondroplasia caused by a common variant, *FGFR3* c.1138G>A. Figure 1B presents the result of case 1b where the maternally inherited *FGFR3* c.1138G>A variant was detected in a single EVT. ADO was observed for the normal allele (ADO: 50%). The finding was confirmed by CVS and the affected newborn child had achondroplasia.

### 3.2 Diastrophic dysplasia (AR skeletal disorders, family-specific variants)

**FIGURE 2:**
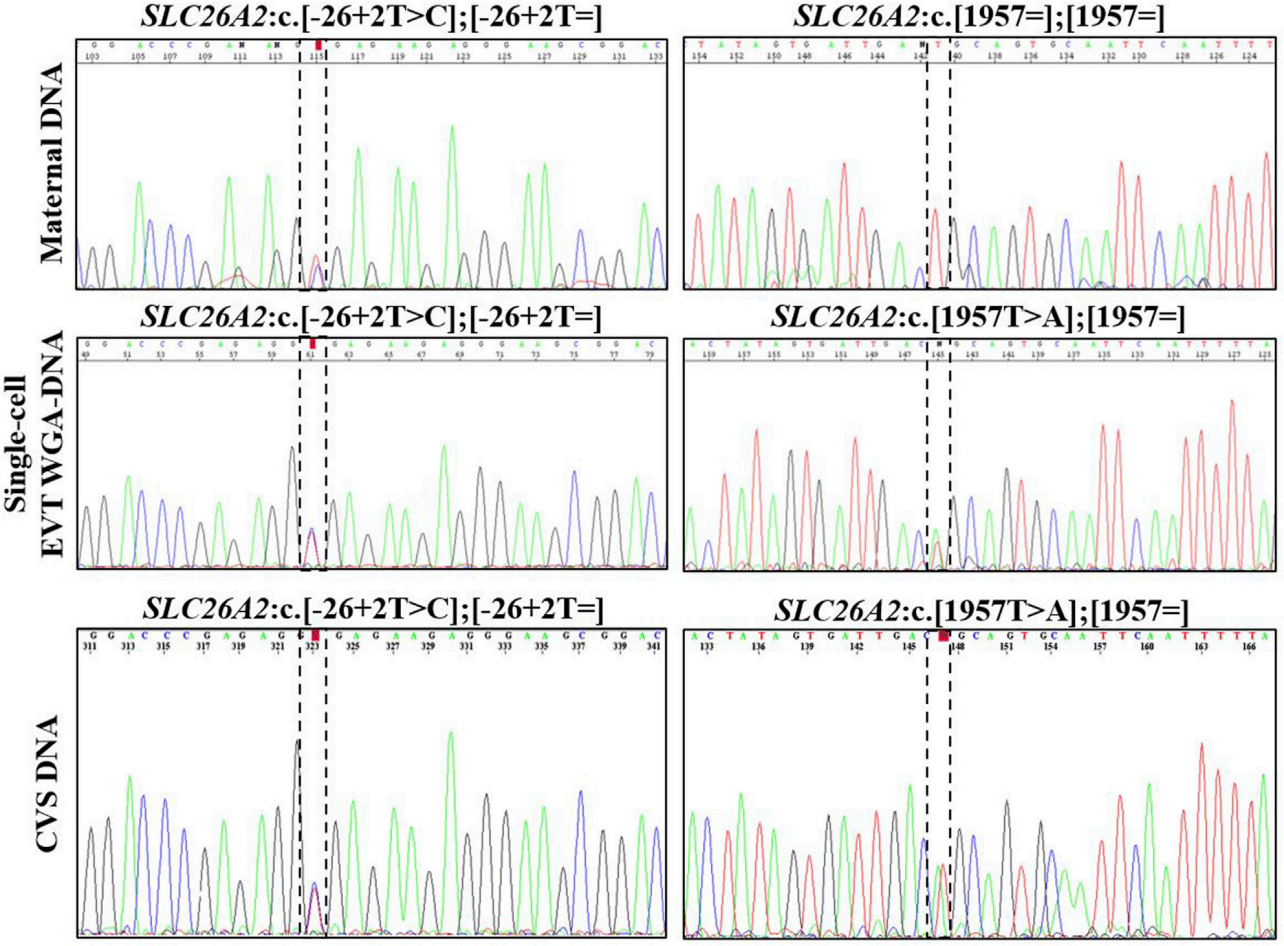
Cell-based NIPT for diastrophic dysplasia. Case 2 sanger sequencing result of maternal DNA maternal DNA, single-cell EVTA WGA-DNA, and CVS DNA. The Sanger sequencing result at the left column covers the region of the maternal variant, *SLC26A2* c.-26 + 2T>C. The column to the right shows the Sanger sequencing result for the paternal variant, *SLC26A* c. 1957T>A. The variant positions are marked by the vertical dotted boxes. The cell-based NIPT result shows that the fetus is compound heterozygote for the parental inherited variants associated with autosomal recessive diastrophic dysplasia.

In case 2, both parents were unaffected carriers of pathogenic variants in *SLC26A2*, associated with AR diastrophic dysplasia. The analysis for the parental diastrophic dysplasia *SLC26A2* c.-26 + 2T>C and c.1957T>A variants was conducted on nine EVTs (Figure 2). For *SLC26A2* c.-26 + 2T>C, PCR product of eight single-cell WGA-DNA and a WGA-DNA pool of those were sequenced, while amplification of the PCR product failed for one EVT. The normal allele was detected in 7/8 (ADO: 12.5%) EVTs and the variant allele c.-26 + 2T>C was detected in 6/8 (ADO: 25.0%) EVTs, consistent with heterozygosity for the variant. Both alleles were detected in the WGA-DNA pool (Figure 2). For *SLC26A2* c.1957T>A, a total of seven single-cell WGA products and a pool of those were sequenced, while two single-cell WGA products failed to amplify the region. The normal allele, c.1957T was detected in 3/7 (ADO: 57.1%) EVTs and the variant allele, c.1957T>A was detected in 7/7 (ADO: 0.0%) EVTs. Both alleles were detected in the WGA-DNA pool. These cell-based NIPT results showed that the fetus was compound heterozygote for the parental *SLC26A2* variants associated with diastrophic dysplasia, in concordance with the invasive test result.

### 3.3 Cystic fibrosis (AR multiorgan disorder, common variants)

**FIGURE 3:**
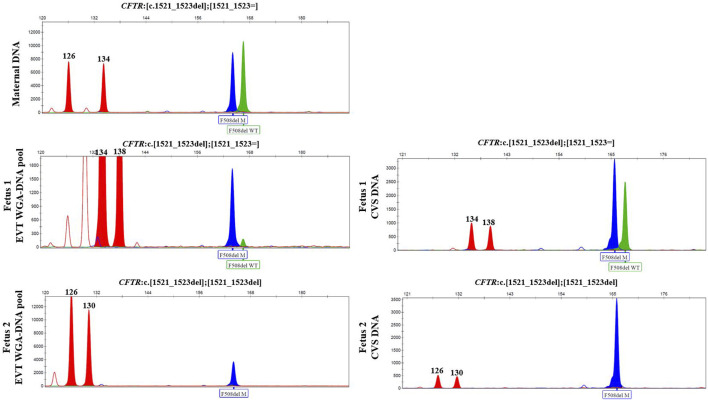
Cell-based NIPT for cystic fibrosis in a twin pregnancy. Both the mother (upper panel) and the father (*data not shown*) were carriers of *CFTR* F508del. The result of two EVTs from fetus 1 detected both the F508del variant allele (blue) and the F508 normal allele (green), which indicate that fetus 1 is an unaffected carrier of cystic fibrosis. Three EVTs from fetus 2 detected only the variant allele, indicating that fetus 2 is affected and the coming child will develop cystic fibrosis. The red marker is a short-tandem repeat marker (D3S1358) at chromosome 3 used to for sample identification (i.e., not for haplotyping).

In case 3, cell-based NIPT was applied to test a dichorionic twin pregnancy where the fetuses were at risk of cystic fibrosis, as both parents carried NM_000492.3(*CFTR*):c.1521_1523del. The result is shown in Figure 3. The result showed that fetus 1 was an unaffected carrier of cystic fibrosis, as both the normal- and the variant allele were detected in the WGA-DNA pool of two EVTs (ADO: 0.0%). In fetus 2, the cell-based NIPT result was based on three EVTs, and only the variant allele was detected (ADO: 0.0%). This result implied that fetus 2 was affected and would develop cystic fibrosis. These results agreed with those of invasive testing.

### 3.4 Duchenne muscular dystrophy (XL muscular wasting disease, a family-specific variant)

**FIGURE 4:**
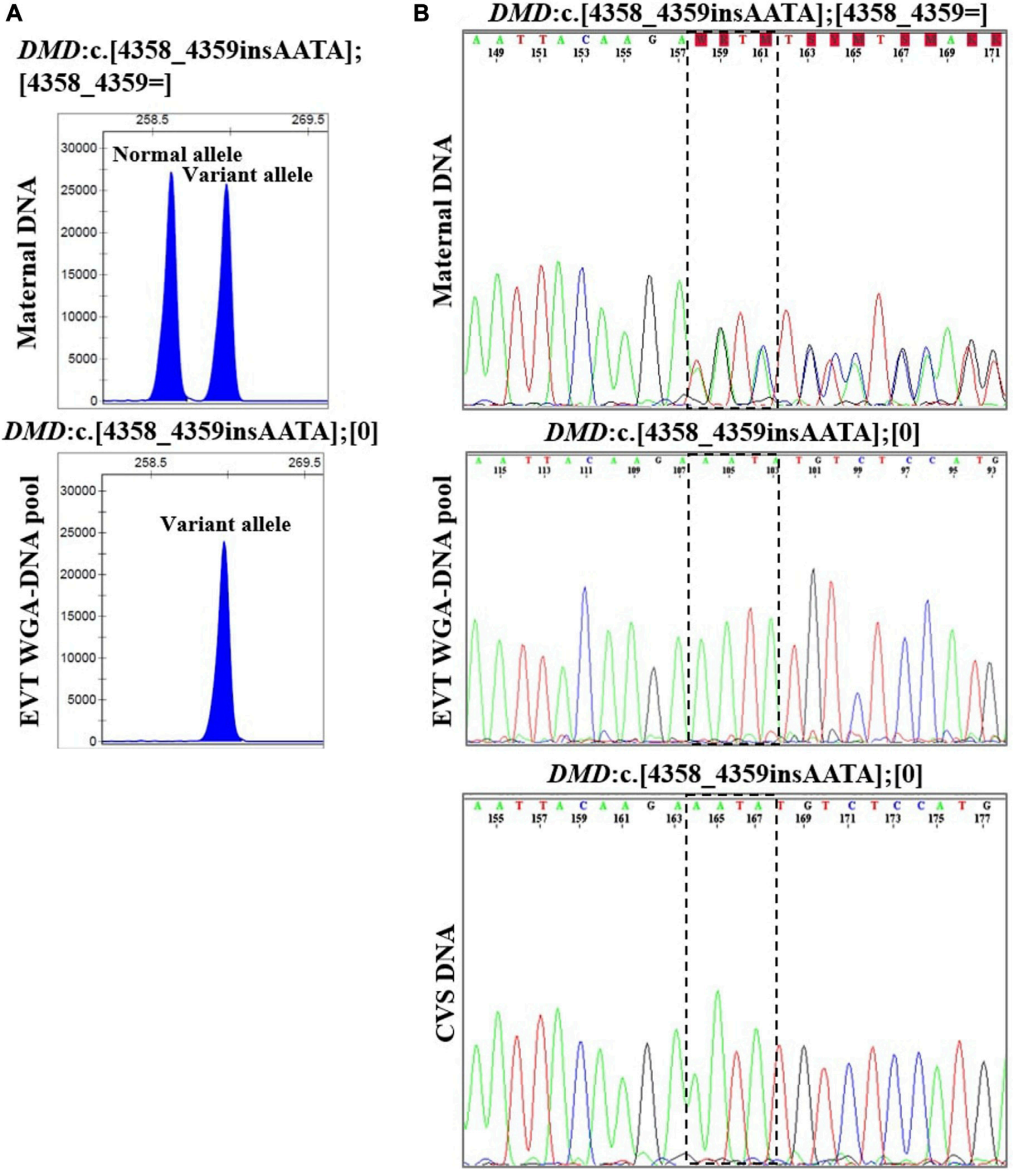
Cell-based NIPT for Duchenne muscular dystrophy. The results shows the detection of a maternally inherited *DMD* variant, c.4358_4359insAATA by direct variant analysis using both fragment length analysis and Sanger sequencing in case 4. **(A)** Direct variant analysis by fragment length analysis of the maternally inherited *DMD* c.4358_4349insAATA. The amplicon representing the normal allele is 260 basepairs and the amplicon representing the variant allele with the 4 basepair insertion is 264 basepairs. The maternal DNA profile shows both a normal allele and a variant allele. The result of the EVT WGA-DNA pool shows the presence of the variant allele with the 4 basepair insertion in the male fetus, indicating that the coming child will develop Duchenne muscular dystrophy. **(B)** Sanger sequencing result of the *DMD* c.4358_4349insAATA variant. The maternal DNA showed presence of both a normal- and a variant allele. The EVT WGA-DNA pool showed a single sequence in the male fetus with the presence of the *DMD* c.4358_4349insAATA variant. The cell-based NIPT result was corroborated by the CVS result.

In case 4, the pregnant woman was an unaffected carrier of XL Duchenne muscular dystrophy. The presence of the maternally inherited variant in *DMD,* c.4358_4359insAATA, was studied using cell-based NIPT using both fragment length analysis and Sanger sequencing. The result is shown in Figure 4. In two EVTs, short tandem repeat analysis including two Y-based loci and the sex determining marker, Amelogenin, was consistent with a male fetus. The results of both fragment length analysis and Sanger sequencing showed the presence of the maternally inherited variant in *DMD,* c. 4358_4359insAATA in the male fetus (ADO: 0.0%). The cell-based NIPT result indicated that the fetus was affected, and this was consistent with the invasive test result.

### 3.5 Myotonic dystrophy type 1 (AD muscle wasting disorder caused by repeat expansion)

**FIGURE 5:**
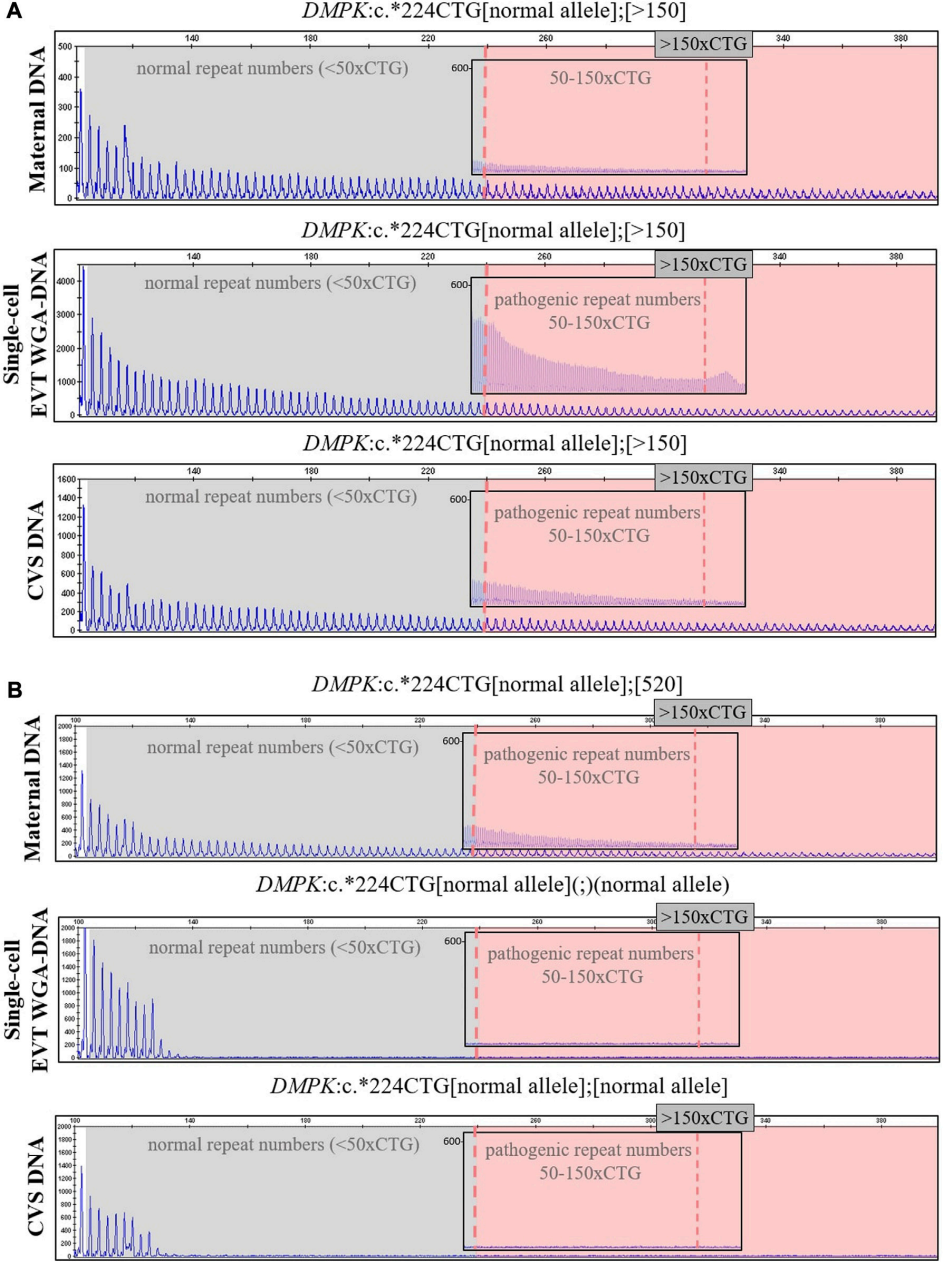
Cell-based NIPT for myotonic dystrophy type 1. The analysis was done by 3′ repeat-primed PCR for detection of the trinucleotide CTG-repeat in DMPK in cases 5a and 5b. The first vertical dotted line marks 50 CTG-repeat and the second verticale dotted line in the insert marks 150 CTG-repeats. **(A)** In case 5a, the single-cell EVT WGA-DNA result shows the presence of a repeat expansion allele with more than 150-CTG repeats. This repeat length marks the pathogenic area associated with the development of myotonic dysotrphy type 1. The result was in concordance with the result of CVS DNA. **(B)** In case 5b, the single-cell EVT WGA-DNA result detected only the normal *DMPK* allele, indicating that the fetus was unaffected. This result was in concordance with the result of CVS DNA.

**FIGURE 6:**
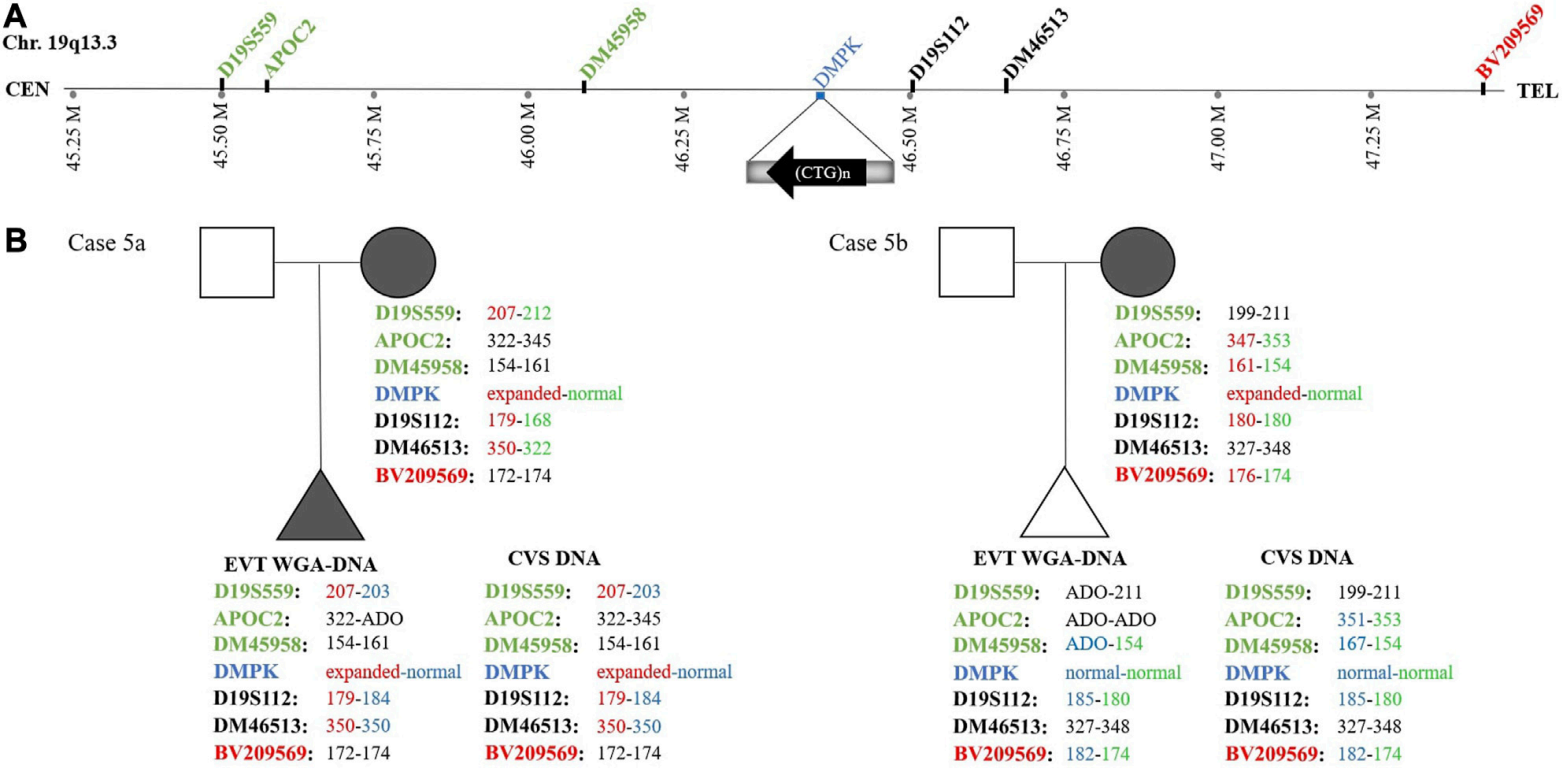
Haplotyping for myotonic dystrophy type 1 analysis of cases 5a and 5b. **(A)** Representation of the location of the short tandem repeat markers relative to the CTG-repeat expansion in the *DMPK* gene. **(B)** The family trees of cases 5a and 5b showing the results of the detected short tandem repeat markers. In case 5a, two fully informative (D19S559 and D19S112) markers and one semi-informative (DM46513) marker show the presence of the expanded *DMPK* allele in the fetus. In case 5b, one fully informative- (BV209569) and one semi-informative (DM45958) marker show the presence of the maternal normal *DMPK* allele in the fetus.

In case 5a, the pregnant woman carried an expanded *DMPK* allele, causing Myotonic dystrophy type 1. Eight EVTs were isolated, and DMPK RP-PCR was performed using single cell WGA-DNA or a WGA-DNA pool as template. For single cell analysis, the expanded allele was detected in 6/8 EVTs (ADO: 25.0%). Figure 5A shows the detection of the repeat expansion in maternal DNA, in a single EVT and the CVS for comparison. The detected repeat pattern exceeded 150 CTG repeats, which is categorized as a full expansion known to be the pathogenic. For this family we also conducted haplotyping analysis using six STR markers closely linked to the *DMPK* repeat expansion (Figure 6; Supplementary Figure S1). Two fully informative STR markers (D19S112 and D19S559), i.e., markers where the parental alleles could be distinguished by different repeat numbers, confirmed the inheritance of the maternal expanded repeat allele (Figure 6). ADO was seen in one out of 11 expected alleles (9.1%), as compared to the profile obtained from CVS DNA (Supplementary Figure S1). The cell-based NIPT result showed that the fetus had inherited a full-expansion in the *DMPK* gene, which will lead to development of myotonic dystrophy type 1.

In case 5b, the pregnant woman had an expanded *DMPK* allele with approximately 520 repeats. The genetic analysis of three EVTs did not detect a repeat expansion in *DMPK* (Figure 5B). This result was supported by haplotyping, where one fully informative STR marker (BV209569) and one semi-informative marker (DM45958) supported the inheritance of the maternal normal allele (Figure 6; Supplementary Figure S1). Compared to the alleles detected on CVS DNA, ADO was observed for three out of 12 alleles (25.0%). Together these cell-based NIPT results indicated an unaffected fetus in concordance with invasive testing.

## 4 Discussion

NIPT using trophoblasts in maternal blood provide an uncontaminated source of fetal DNA, which allows for direct detection independent of maternal or paternal origin of the variant(s). In this study, EVTs were enriched from maternal blood using a combination of mesenchymal and epithelial markers as previously described (Hatt et al., 2014a). The present study demonstrates how circulating EVTs can be used to detect different types of variants associated with AD, AR, XL and repeat expansion disorders. For all seven cases presented in this study, there was concordance between the results of cell-based NIPT and invasive testing; however, we also acknowledge some of the challenges of single cell analysis in NIPT; these are summarized in Table 2.

**TABLE 2:** Clinical interpretation of cell-based NIPT results for monogenic disorders based on mode of inheritance.

Mode of inheritance	Allele detection cell-based NIPT	Clinical interpretation of results	Actions required	Cases in this study
Autosomal dominant	Variant	Affected, ADO of normal allele	Invasive confirmatory testing if TOP is considered	1a, 1b, 2a
	Variant + normal	Affected	Invasive confirmatory testing if TOP is considered	
	Normal	Unaffected OR ADO of variant allele	Haplotyping to avoid false-negative	5b
Autosomal recessive (parents carry different variants)	Maternal variant + paternal variant	Affected	Invasive confirmatory testing if TOP is considered	3
	Only maternal or paternal variant	Unaffected OR ADO of variant allele	Haplotyping to avoid false-negative	
	Normal	Unaffected OR ADO of both variant alleles	Haplotyping to avoid false-negative	
Autosomal recessive (parents carry same variants)	Variant	Affected OR ADO of normal allele	Haplotyping to avoid false-positive and/or invasive confirmatory testing if TOP is considered	3 (fetus 2)
	Variant + normal	Unaffected carrier	None	3 (fetus 1)
	Normal	Unaffected, carrier status unknown	None	
X-linked				
Female fetus (X-linked recessive)	Female fetus	Unaffected, carrier status unknown	None	
Female fetus (X-linked dominant)	Variant	Affected, ADO of normal allele	Invasive confirmatory testing if TOP is considered	
	Variant + normal	Affected	Invasive confirmatory testing if TOP is considered	
	Normal	Unaffected OR ADO of variant allele	Haplotyping to avoid false-negative	
Male fetus (recessive or dominant)	Variant	Affected	Invasive confirmatory testing if TOP is considered	4
	Normal	Unaffected	None	

TOP, termination of pregnancy; ADO, allelic dropout.

### 4.1 Cell-based noninvasive prenatal testing of variants associated with autosomal dominant disorders

Cell-free NIPT for variants associated with AD disorders has been developed for detection of paternally and *de novo* variants, while the status of maternally inherited variants relies on molecular counting techniques to determine the fetal allele frequency, necessitating paternal or proband samples (Jenkins et al., 2018). In the present study, two cases of *FGFR3*-related disorders, cases 1a and 1b, were tested using cell-based NIPT. In case 1a, the father was affected by hypochondroplasia caused by a single nucleotide variant in *FGFR3*. Cell-based NIPT detected the paternally inherited disease-causing *FGFR3* variant in one EVT. ADO, i.e., failure to detect one allele, was observed for the maternal allele.

In case 1b, the pregnant woman was affected by achondroplasia. Cell-based NIPT detected the maternally inherited disease-causing *FGFR3* variant, while ADO was observed for the paternal allele. In both cases, cell-based NIPT correctly identified the fetuses’ status of the variant alleles. However, in cases where only a normal allele is detected, the risk of ADO of the variant allele must be considered. These results indicate that direct analysis of variants associated with AD disorders cannot stand alone to diagnose an unaffected pregnancy. We have previously shown how haplotyping increases the diagnostic yield of cell-based NIPT to confirm an unaffected pregnancy following preimplantation genetic testing (PGT) (Toft et al., 2021). In cases at high-risk (50%) of an AD disorder, haplotyping using STR or single nucleotide polymorphisms (SNPs) flanking the gene of interest could also be included in the analysis. These markers could support the diagnosis of an unaffected fetus when the cell-based NIPT result detects only the normal allele(s), see Table 2.

### 4.2 Cell-based noninvasive prenatal testing of variants associated with autosomal recessive disorders

The interpretation of cell-based NIPT for variants associated with AR disorders varies, depending on whether the parents carry different- or the same variants (see Table 2). Here, we present both situations in cases 2 and 3. In the first case (case 2), the parents carried different disease-causing variants in *SLC26A2*, associated with the development of AR diastrophic dysplasia. Cell-based NIPT gave an accurate result of an affected fetus that was compound heterozygote for the parental *SLC26A2* variants. Detection of both the variant and the normal alleles at both sites of interests gave a clear interpretation of the result. In cases where the parents carry different disease-causing variants, ADO of one- or both variant alleles could, however, lead to a false-negative-result. Hence, to diagnose an unaffected fetus, a negative cell-based NIPT result for AR disorders, where the parents carry different disease-causing variants, should also be supported by haplotyping (Table 2).

The clinical interpretation of cell-based NIPT results for an AR disorder, where the parents carry the same pathogenic variant, is different (see Table 2). In these cases, ADO of a normal allele leads to a false-positive result. Detection of a normal allele would oppositely be sufficient to diagnose an unaffected pregnancy. In case 3, which was a twin pregnancy, both parents were carriers of the common cystic fibrosis variant frequently known as delta508. The cystic fibrosis analysis showed that one of the fetuses was homozygote for the variant, and will develop the disease, while the other was heterozygote for delta508, and will not. Although this case is complex as it seeks to diagnose a twin pregnancy, it demonstrates the potential of cell-based NIPT for prenatal testing of cystic fibrosis, where delta508 is a common variant constituting 88% of the *CFTR* variant alleles in the Danish population (Schwartz et al., 1990; Schwartz et al., 1992; de Boeck et al., 2014). In Denmark, there is currently no universal carrier screening program for monogenic disorders, although introduction of prenatal screening for cystic fibrosis is being debated (Smed et al., 2021). In this context, cell-based noninvasive prenatal screening for cystic fibrosis has recently been developed to state the fetal status for the 50 most common cystic fibrosis variants without the need of a partner sample (Jeppesen et al., 2023). Together with the present study, these results suggest that cell-based NIPT for AR disorders can be developed either tailored for each family or as a universal prenatal screening for common disease-causing variants early in pregnancy.

### 4.3 Cell-based noninvasive prenatal testing of variants associated with X-linked disorders

Cell-based NIPT of XL disorders depends on the fetal sex, which was already identified by short tandem repeat markers at the sex chromosomes. For female fetuses, the clinical interpretation of cell-based NIPT results depends on the pattern of inheritance which may be X-linked recessive (XLR) or dominant (XLD) (see Table 2). Female fetuses will be unaffected for diseases following a XLR pattern of inheritance, while for XLD disorders, the interpretation of the cell-based NIPT result follow the reasoning for AD disorders. In between exist the cases with variable penetrance and hence a variable phenotype for female carriers, which requires thorough counseling by a clinical geneticist. For male fetuses, cell-based NIPT for XL disorders can provide a direct diagnosis by detection of either a normal- or variant allele. This was shown in case 4, where cell-based NIPT correctly stated a male fetus that had inherited the maternal variant allele causing the development of XLR Duchenne Muscular Dystrophy.

### 4.4 Cell-based noninvasive prenatal testing of repeat expansion disorders

Prenatal diagnostic testing by direct variant analysis is achieved by repeat-primed PCR (RP-PCR) of the trinucleotide repeat expansion. In cases where the parents share the same repeat number of the normal alleles, a normal test result should be supported by haplotyping to avoid misdiagnosis due to ADO of the expanded allele (Kamsteeg et al., 2012). This applies for invasive-as well as noninvasive prenatal testing. Noninvasive testing using fetal cell-free DNA for repeat expansion disorders has solely been based on haplotyping, because the majority of fetal cell-free DNA fragments are simply too short for direct analysis of the repeat expansions (Liautard-Haag et al., 2022). However, in molecular diagnostics of repeat expansion disorders, haplotyping alone is insufficient to predict the phenotype of the coming child, as the repeat number is susceptible to expanding from one generation to the next. The existence of long fetal cell-free DNA fragments of more than 500 base pairs has recently been described, still, the potential clinical utility in noninvasive testing of monogenic disorders is yet to be explored (Yu et al., 2021).

In the present study, we used cell-based NIPT in two cases, 5a and 5b, to test for myotonic dystrophy type 1 caused by a CTG-repeat expansion in *DMPK*. In case 5a, the pregnant woman carried an *DMPK* allele with more than 150 CTG-repeats. Direct variant analysis of WGA-DNA from EVTs clearly detected the expanded maternally inherited *DMPK* allele, indicating that the coming child would develop myotonic dystrophy type 1. The cell-based NIPT result agreed with the result of invasive testing. In the second case, the mother carried a full expansion *DMPK* allele with more than 500 CTG-repeats. The cell-based result detected only the normal allele(s), indicating an unaffected fetus. The negative cell-based NIPT result was supported by haplotyping where one fully informative and one semi-informative short tandem repeat marker confirmed the presence of the normal maternally inherited *DMPK* allele. The cell-based NIPT result agreed with that of invasive testing. Hence, this specific case (5b) exemplify how haplotyping can support the diagnosis of an unaffected pregnancy. Cell-based NIPT for repeat expansion disorders has previously been demonstrated by Toft et al. (2021) in two cases to confirm an unaffected fetus following PGT for myotonic dystrophy type 1 and Fragile-X syndrome, the latter caused by repeat expansions in *FMR1* on chromosome X. These combined results demonstrate the potential of circulating EVTs for NIPT of repeat expansion disorders.

### 4.5 Interpretation of cell-based noninvasive prenatal testing for monogenic disorders and future perspectives

This study compares the results of cell-based NIPT for monogenic disorders with those of invasive testing, which is the current gold standard for prenatal diagnostics. In contrast to invasive sampling, cell-based NIPT is without risk of procedure-related loss. This has shown to be the primary reason for declining invasive testing among women undergoing *in vitro fertilization* and preimplantation genetic testing (Toft et al., 2022). Although circulating EVTs deliver an intact fetal genome, the subsequent whole-genome amplification (WGA) procedure results in fragmentation of the DNA, sometimes resulting in failure to amplify one allele (ADO) (van der Plaetsen et al., 2017). The cell-based NIPT results of the presented cases together demonstrate how the consequence of ADO varies - and can be managed - depending on the disease inheritance mode and parental variants. We showed how linked markers (SNPs or STRs) can be useful to support a negative cell-based test-result showing an unaffected fetus. Further validation studies are required to determine the accuracy of cell-based NIPT for monogenic disorders with respect to the risk of false-positive and false-negative results caused by failure to amplify either the variant- or normal allele.

Hanson et al. (2022) recently summarized the limitations of cell-free DNA in prenatal diagnostics dependent on mode of inheritance. Our study shows how the clinical interpretation of the cell-based NIPT result also depends on this, although in different ways. For detection of paternally-inherited and *de novo* variants, testing of fetal cell-free DNA may offer a simpler alternative to invasive testing (especially in the end of second trimester when the fetal cell-free DNA fraction is high) compared to isolation of circulating trophoblasts. On the other hand, cell-based NIPT may be advantageous to determine the status for the maternally inherited allele, where cell-free NIPT depends on massive parallel sequencing for relative haplotype- or mutation dosage analysis. The clinical interpretation of a cell-based NIPT result and the limitations of the method are summarized in Table 2. These implications and how to overcome them must be considered in future research and in the clinical practice.

In a recent study, Hatt et al. (2023) aimed to explore how cell-based NIPT performed compared to invasive testing (CVS) and cell-free NIPT in detecting aneuploidy and copy number variants. These data showed that failure to obtain a result occurred more often when using cell-based NIPT (7.8%) as compared to cell-free NIPT (2.8%) but redraw reduced the failure rate of cell-based NIPT to 2.0% (Hatt et al., 2023). As for other prenatal (screening) tests, the cell-based NIPT test limitations and residual risk must be communicated in both pre and post-test counseling.

In summary, cell-based NIPT is a promising tool for the detection of both paternally- and maternally inherited disease-causing variants including repeat expansion disorders. Additional testing strategies are, however, needed to reduce the risk of misdiagnosis. We conclude that cell-based NIPT may be a safe prenatal testing option for monogenic disorders if used correctly in clinical practice.

## Data Availability

The original contributions presented in the study are included in the article/Supplementary Material, further inquiries can be directed to the corresponding author.

## Nomenclature

The sequence variant nomenclature was adopted from Human Genome Variation Society (HGVS) recommendations for the description of sequence variants (den Dunnen et al., 2016). Soft parentheses () were used for results where the analysis detects one variant [e.g., (c.1620C>G)], suggesting both alleles (chromosomes) contain this variant, but allelic dropout, i.e., the failure to amplify one alle, could not be excluded.

## References

[B1] Chang L. Zhu X. Li R. Wu H. Chen W. Chen J. (2021). A novel method for noninvasive diagnosis of monogenic diseases from circulating fetal cells. Prenat. Diagn 41, 400–408. 10.1002/pd.5796 32673403

[B2] Chitty L. S. Griffin D. R. Meaney C. Barrett A. Khalil A. Pajkrt E. (2011). New aids for the non-invasive prenatal diagnosis of achondroplasia: dysmorphic features, charts of fetal size and molecular confirmation using cell-free fetal DNA in maternal plasma. Ultrasound Obstetrics Gynecol. 37, 283–289. 10.1002/uog.8893 21105021

[B3] de Boeck K. Zolin A. Cuppens H. Olesen H. Viviani L. (2014). The relative frequency of CFTR mutation classes in European patients with cystic fibrosis. J. Cyst. Fibros. 13, 403–409. 10.1016/j.jcf.2013.12.003 24440181

[B4] den Dunnen J. T. Dalgleish R. Maglott D. R. Hart R. K. Greenblatt M. S. Mcgowan-Jordan J. (2016). HGVS recommendations for the description of sequence variants: 2016 update. Hum. Mutat. 37, 564–569. 10.1002/HUMU.22981 26931183

[B5] Hanson B. Scotchman E. Chitty L. S. Chandler N. J. (2022). Non-invasive prenatal diagnosis (NIPD): how analysis of cell-free DNA in maternal plasma has changed prenatal diagnosis for monogenic disorders. Clin. Sci. (Lond) 136, 1615–1629. 10.1042/CS20210380 36383187 PMC9670272

[B6] Hatt L. Brinch M. Singh R. Møller K. Lauridsen R. H. Schlütter J. M. (2014a). A new marker set that identifies fetal cells in maternal circulation with high specificity. Prenat. Diagn 34, 1066–1072. 10.1002/pd.4429 24912661

[B7] Hatt L. Brinch M. Singh R. Møller K. Lauridsen R. H. Uldbjerg N. (2014b). Characterization of fetal cells from the maternal circulation by microarray gene expression analysis - could the extravillous trophoblasts be a target for future cell-based non-invasive prenatal diagnosis? Fetal Diagn Ther. 35, 218–227. 10.1159/000356073 24217417

[B8] Hatt L. Ravn K. Dahl Jeppesen L. Hestbek Nicolaisen B. Baasch Christensen I. Singh R. (2023). How does cell-based non-invasive prenatal test (NIPT) perform against chorionic villus sampling and cell-free NIPT in detecting trisomies and copy number variations? A clinical study from Denmark. Prenat. Diagn 43, 854–864. 10.1002/pd.6387 37199490

[B9] Hatt L. Singh R. Christensen R. Ravn K. Christensen I. B. Jeppesen L. D. (2020). Cell-based noninvasive prenatal testing (cbNIPT) detects pathogenic copy number variations. Clin. Case Rep. 8, 2561–2567. 10.1002/ccr3.3211 33363780 PMC7752386

[B10] Hill M. Compton C. Karunaratna M. Lewis C. Chitty L. (2014). Client views and attitudes to non-invasive prenatal diagnosis for sickle cell disease, thalassaemia and cystic fibrosis. J. Genet. Couns. 23, 1012–1021. 10.1007/s10897-014-9725-4 24788196

[B11] Hill M. Johnson J. A. Langlois S. Lee H. Winsor S. Dineley B. (2016). Preferences for prenatal tests for down syndrome: an international comparison of the views of pregnant women and health professionals. Eur. J. Hum. Genet. 24, 968–975. 10.1038/ejhg.2015.249 26577044 PMC5070900

[B12] Jenkins L. A. Deans Z. C. Lewis C. Allen S. (2018). Delivering an accredited non-invasive prenatal diagnosis service for monogenic disorders and recommendations for best practice. Prenat. Diagn 38, 44–51. 10.1002/pd.5197 29266293

[B13] Jeppesen L. D. Hatt L. Singh R. Ravn K. Kølvraa M. Schelde P. (2021). Cell-based non-invasive prenatal diagnosis in a pregnancy at risk of cystic fibrosis. Prenat. Diagn 41, 234–240. 10.1002/pd.5861 33150588

[B14] Jeppesen L. D. Lildballe D. L. Hatt L. Hedegaard J. Singh R. Frisk Toft C. L. (2023). Noninvasive prenatal screening for cystic fibrosis using circulating trophoblasts: detection of the 50 most common disease-causing variants. Prenat. Diagn 43, 3–13. 10.1002/pd.6276 36447355 PMC10107343

[B15] Kamsteeg E. J. Kress W. Catalli C. Hertz J. M. Witsch-Baumgartner M. Buckley M. F. (2012). Best practice guidelines and recommendations on the molecular diagnosis of myotonic dystrophy types 1 and 2. Eur. J. Hum. Genet. 20, 1203–1208. 10.1038/ejhg.2012.108 22643181 PMC3499739

[B16] Lian M. Rajan-Babu I.-S. Singh K. Lee C. G. Law H.-Y. Chong S. S. (2015). Efficient and highly sensitive screen for myotonic dystrophy type 1 using a one-step triplet-primed PCR and melting curve assay. J. Mol. Diagn 17, 128–135. 10.1016/j.jmoldx.2014.10.001 25684273

[B17] Lian M. Zhao M. Lee C. G. Chong S. S. (2017). Single-tube dodecaplex PCR panel of polymorphic microsatellite markers closely linked to the DMPK CTG repeat for preimplantation genetic diagnosis of myotonic dystrophy type 1. Clin. Chem. 63, 1127–1140. 10.1373/clinchem.2017.271528 28428361

[B18] Liautard-Haag C. Durif G. VanGoethem C. Baux D. Louis A. Cayrefourcq L. (2022). Noninvasive prenatal diagnosis of genetic diseases induced by triplet repeat expansion by linked read haplotyping and Bayesian approach. Sci. Rep. 12, 11423. 10.1038/s41598-022-15307-2 35794169 PMC9259573

[B19] Lund I. C. B. Becher N. Petersen O. B. Hill M. Chitty L. Vogel I. (2018). Preferences for prenatal testing among pregnant women, partners and health professionals. Dan. Med. J. 65, A5486. 29726320

[B20] Mirkin S. M. (2007). Expandable DNA repeats and human disease. Nature 447, 932–940. 10.1038/nature05977 17581576

[B21] Mohan P. Lemoine J. Trotter C. Rakova I. Billings P. Peacock S. (2022). Clinical experience with non-invasive prenatal screening for single-gene disorders. Ultrasound Obstetrics Gynecol. 59, 33–39. 10.1002/uog.23756 PMC9302116 34358384

[B22] Mouawia H. Saker A. Jais J. P. Benachi A. Bussières L. Lacour B. (2012). Circulating trophoblastic cells provide genetic diagnosis in 63 fetuses at risk for cystic fibrosis or spinal muscular atrophy. Reprod. Biomed. Online 25, 508–520. 10.1016/j.rbmo.2012.08.002 23000084

[B23] Ravn K. Singh R. Hatt L. Kølvraa M. Schelde P. Vogel I. (2020). The number of circulating fetal extravillous trophoblasts varies from gestational week 6 to 20. Reprod. Sci. 27, 2170–2174. 10.1007/s43032-020-00243-1 32602048 PMC7593292

[B24] Saker A. Benachi A. Bonnefont J. P. Munnich A. Dumez Y. Lacour B. (2006). Genetic characterisation of circulating fetal cells allows non-invasive prenatal diagnosis of cystic fibrosis. Prenat. Diagn 26, 906–916. 10.1002/pd.1524 16832834

[B25] Salomon L. J. Sotiriadis A. Wulff C. B. Odibo A. Akolekar R. (2019). Risk of miscarriage following amniocentesis or chorionic villus sampling: systematic review of literature and updated meta-analysis. Ultrasound Obstetrics Gynecol. 54, 442–451. 10.1002/uog.20353 31124209

[B26] Schwartz M. Brandt N. Koch C. Lanng S. Schiøtz P. (1992). Genetic analysis of cystic fibrosis in Denmark. Implications for genetic counselling, carrier diagnosis and prenatal diagnosis. Acta Paediatr. 81, 522–526. 10.1111/j.1651-2227.1992.tb12287.x 1392366

[B27] Schwartz M. Johansen H. K. Koch C. Brandt N. J. (1990). Frequency of the ΔF508 mutation on cystic fibrosis chromosomes in Denmark. Hum. Genet. 85, 427–428. 10.1007/BF02428297 2210762

[B28] Scotchman E. Chandler N. J. Mellis R. Chitty L. S. (2020a). Noninvasive prenatal diagnosis of single-gene diseases: the next frontier. Clin. Chem. 66, 53–60. 10.1373/clinchem.2019.304238 31843868

[B29] Scotchman E. Shaw J. Paternoster B. Chandler N. Chitty L. S. (2020b). Non-invasive prenatal diagnosis and screening for monogenic disorders. Eur. J. Obstetrics Gynecol. Reproductive Biol. 253, 320–327. 10.1016/j.ejogrb.2020.08.001 32907778

[B30] Singh R. Hatt L. Ravn K. Vogel I. Petersen O. B. Uldbjerg N. (2017). Fetal cells in maternal blood for prenatal diagnosis: A love story rekindled. Biomark. Med. 11, 705–710. 10.2217/bmm-2017-0055 28617034

[B31] Smed V. M. Bennike O. Petersen B. Gerdes A.-M. Diness B. R. Roos L. S. (2021). Genetisk screening af kommende foraeldre. Ugeskr. Læg. V12200933, 183.

[B33] Toft C. L. F. Diemer T. Ingerslev H. J. Pedersen I. S. Adrian S. W. Kesmodel U. S. (2022). Patients’ choices and opinions on chorionic villous sampling and non-invasive alternatives for prenatal testing following preimplantation genetic testing for hereditary disorders: A cross-sectional questionnaire study. Prenat. Diagn 42, 212–225. 10.1002/pd.6088 34997771

[B34] Toft C. L. F. Ingerslev H. J. Kesmodel U. S. Hatt L. Singh R. Ravn K. (2021). Cell-based non-invasive prenatal testing for monogenic disorders: confirmation of unaffected fetuses following preimplantation genetic testing. J. Assist. Reprod. Genet. 38, 1959–1970. 10.1007/s10815-021-02104-5 33677749 PMC8417213

[B35] van der Plaetsen A. S. Deleye L. Cornelis S. Tilleman L. van Nieuwerburgh F. Deforce D. (2017). STR profiling and copy number variation analysis on single, preserved cells using current Whole Genome Amplification methods. Sci. Rep. 7, 17189. 10.1038/s41598-017-17525-5 29215049 PMC5719346

[B36] Wulff C. B. Gerds T. A. Rode L. Ekelund C. K. Petersen O. B. Tabor A. (2016). Risk of fetal loss associated with invasive testing following combined first-trimester screening for down syndrome: A national cohort of 147 987 singleton pregnancies. Ultrasound Obstetrics Gynecol. 47, 38–44. 10.1002/uog.15820 26581188

[B37] Yu S. C. Y. Jiang P. Peng W. Cheng S. H. Cheung Y. T. T. Tse O. Y. O. (2021). Single-molecule sequencing reveals a large population of long cell-free DNA molecules in maternal plasma. 10.1073/pnas.2114937118 PMC8685924 34873045

[B38] Zhuo X. Wang Q. Vossaert L. Salman R. Kim A. Van DenIgnatia V. (2021). Use of amplicon-based sequencing for testing fetal identity and monogenic traits with Single Circulating Trophoblast (SCT) as one form of cell-based NIPT. PLoS One 16, e0249695. 10.1371/journal.pone.0249695 33857205 PMC8049273

