# Supplementary material for: Clinical interpretation of cell-based non-invasive prenatal testing for monogenic disorders including repeat expansion disorders: potentials and pitfalls
Source: Front Genet. 2023 Sep 27;14:1188472. doi: 10.3389/fgene.2023.1188472 (PMC10565008; doi:10.3389/fgene.2023.1188472)
Supplement: Supplementary file 1 [file Table1.pdf]

frontiers

Supplementary Material

Clinical interpretation of cell-based noninvasive prenatal testing of monogenic disorders including repeat expansion disorders: Potentials and pitfalls

Line Dahl Jeppesen[1,2], Lotte Hatt[1], Ripudaman Singh[1], Palle Schelde[1], Katarina Ravn[1], Christian Liebst Toft[3,4], Maria Bach Laursen[1], Jakob Hedegaard[1], Inga Baasch Christensen[1], Bolette Hestbek Nicolaisen[1], Lotte Andreasen[5], Lars Henning Pedersen[6,7,8], Ida Vogel[2,6], Dorte L. Lildballe[2,9]

[1]ARCEDI Biotech, Vejle, Denmark

[2]Center for Fetal Diagnostics, Aarhus University, Aarhus, Denmark

[3]Department of Molecular Diagnostics, Aalborg University Hospital, Aalborg, Denmark

[4]Center for Preimplantation Genetic Testing, Aalborg University Hospital, Aalborg, Denmark

[5]Department of Clinical Genetics, Aarhus University Hospital, Denmark

[6]Department of Gynecology and Obstetrics, Aarhus University Hospital, Aarhus, Denmark

[7]Department of Clinical Medicine, Aarhus University, Aarhus Denmark

[8]Department of Biomedicine, Aarhus University, Aarhus Denmark

[9]Department of Molecular Medicine, Aarhus University Hospital, Aarhus, Denmark

*** Correspondence:**
Line Dahl Jeppesen
linedahljeppesen@clin.au.dk.

1 Supplementary Figures and Tables

1.1 Supplementary Figures

Supplementary Figure 1: (A) Short tandem repeat haplotyping result of case 5a. Two fully informative STR markers (D19S112 and D19S559), i.e. markers where the parental alleles could be distinguished by different repeat numbers, confirmed the inheritance of the maternal expanded repeat allele. Allele drop out, i.e. loss of one allele during PCR amplification, was seen in one out of 11 expected alleles (9.09%), as compared to the profile obtained from CVS DNA. (B) Short tandem repeat haplotyping result of case 5b. One fully informative STR marker (BV209569) and one semi-

informative STR marker (D19S112) were identified. Compared to the alleles detected on CVS DNA, ADO was observed for three out of 12 alleles (25%).

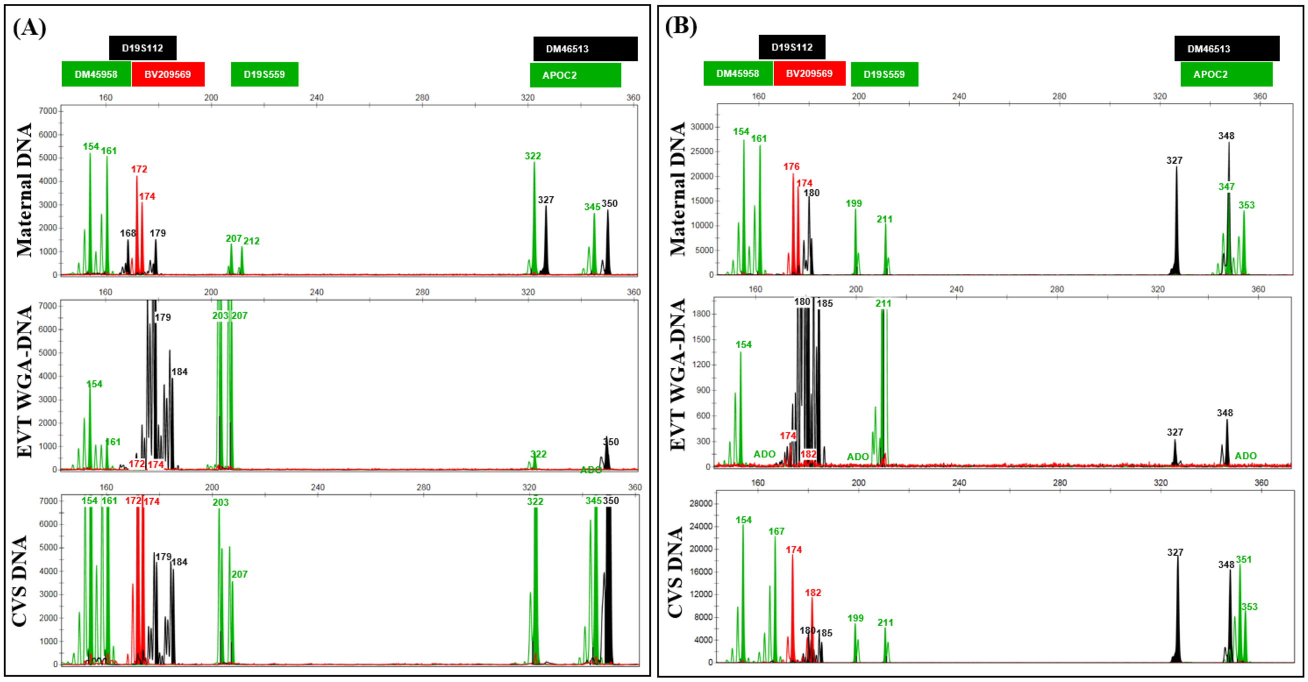

1.2 Supplementary Tables

Supplementary Table 1: Primer details for detection of *FGFR3*-related disorders.

Amplicon	Forward primer	Reverse primer	Final conc. (µM)	Annealing temperature (°C)
***FGFR3*_c.1138G >A_region**	FAM-CCGAGGAGG AGCTGGTGGA	CTGTCGCTTGAGCGGG AAGC	0.2	58
***FGFR3*_c.1620C >G _region**	FAM-GACCTGTCGGACCTGGTGT C	GCAGAAACTCCCGCAG GTTA	2.0	60

Supplementary Table 2: Primer details for detection of Diastrophic Dysplasia.

Amplicon	Forward primer	Reverse primer
***SLC26A2* c.-26+2 T>C**	GCGGTGTCCACCTCAGTCAG	GGGGACCCAGGCAGAATGAC
***SLC26A2* c.1957 T>A**	TGACTCTTGGTGGAATCCAGG	ATCCCTCACAGTGGGATTGC

Supplementary Table 3: Primer details for Duchenne Muscular dystrophy (*DMD*[c.4358_4359insAATA]).

Amplicon	Forward primer	Reverse primer
DMD_c.4358_4359insAATA_region	M13F+TCCTGTGTTGGATGAATGGAA	M13R+AGACACACAGAATAGGCCACA
**M13_sequencing**	TGTAAAACGACGGCCAGT	CAGGAAACAGCTATGACC

Supplementary Table 4: Primer details for detection of Myotonic Dystrophy Type 1

Marker	Forward primer	Reverse primer
DMPK	TACGCATCCGAGTTTGAGACGTGCTGCTGCTGCTG (3' TPF)	FAM-GGCCTGCAGTTTGCCCATCCACGTCAG (FAM-3' R)
**3'-tail**	ATCGAGTCCAGTCGATCTATGC	
**DM45958**	VIC-GCACCTAGAGTGTGTGACTGC	AGCCTTCCCCTAAGACCAGG[a]
**BV209569**	PET-GAGTGTTTCTGTTATTGTGGAGGG	GGATGTTACAACACCATGCTGG[a]
**D19S112**	NED-GGCTCTCTGGTTGCTCATTC	TGGACCCACAGAAACTGAAAGAC
**DM46513**	NED-TAAGTGCTGACCCGGAGGAC	GAGGAAGATAAGAAGCCACTGAGC[a]
**APOC2**	VIC-TAAGAAAGGGACTCAGGGTGC	CACCCTAACTCTAAGCAGAAGCTC[b]
**DM46513**	NED-TAAGTGCTGACCCGGAGGAC	GAGGAAGATAAGAAGCCACTGAGC[a]

[a]GTTT-tail sequence in 5' end, [b]GTTCTT-tail sequence in 5' end
